# How Beauty Determines Gaze! Facial Attractiveness and Gaze Duration in Images of Real World Scenes

**DOI:** 10.1177/2041669516664355

**Published:** 2016-08-23

**Authors:** Helmut Leder, Aleksandra Mitrovic, Jürgen Goller

**Affiliations:** Department of Basic Psychological Research and Research Methods, Faculty of Psychology, University of Vienna, Austria

**Keywords:** eye movements, facial attractiveness, empirical aesthetics, scene perception, visual attention

## Abstract

We showed that the looking time spent on faces is a valid covariate of beauty by testing the relation between facial attractiveness and gaze behavior. We presented natural scenes which always pictured two people, encompassing a wide range of facial attractiveness. Employing measurements of eye movements in a free viewing paradigm, we found a linear relation between facial attractiveness and gaze behavior: The more attractive the face, the longer and the more often it was looked at. In line with evolutionary approaches, the positive relation was particularly pronounced when participants viewed other sex faces.

## Introduction

Facial attractiveness is a pervasive factor in everyday life. Even though people are often not aware of its impact, attractiveness affects our social perception and interactions in various ways (Brand, Bonatsos, D’Orazio, & DeShong, 2012; Dion, Walster, & Berscheid, 1972; Langlois et al., 2000; Tsukiura & Cabeza, 2011). Our attention is bound by attractive people as an automatic effect which is hard to evade (Chen, Liu, & Nakabayashi, 2012; DeWall & Maner, 2008; Sui & Liu, 2009; Valuch, Pflüger, Wallner, Laeng, & Ansorge, 2015). Even newborns and infants show this pattern, which is why the effects of attractiveness on guiding our visual attention are considered inborn and hardwired (Langlois, Ritter, Roggman, & Vaughn, 1991; Slater et al., 1998). The psychological mechanism behind this beauty bias might be as simple as it is convincing: Looking at an attractive face is rewarding and elicits pleasure and positive emotions (Aharon et al., 2001; Cloutier, Heatherton, Whalen, & Kelley, 2008; Hahn & Perrett, 2014; Vartanian, Goel, Lam, Fisher, & Granic, 2013). From this it follows that providing positively toned events might be the main tool of the biologically determined sense of beauty (Darwin, 1859). However, empirically supporting this claim requires showing that facial attractiveness systematically and sensitively affects visual exploration.

Early behavioral effects of beauty were found in studies on face-to-face interactions, in which male participants looked longer at attractive than less attractive women (Fugita, Agle, Newman, & Walfish, 1977; Kleck & Rubenstein, 1975). Similar effects were later found by applying state-of-the-art eye tracking methods in laboratory settings. The gaze has been shown to shift toward the more attractive face when two faces are presented and people are asked to indicate the more attractive face (Shimojo, Simion, Shimojo, & Scheier, 2003). However, in this study, the effect could be confounded by the choice participants had to make and therefore might not only reflect the attractiveness of the face. It is therefore important to implement a free viewing paradigm to exclude possible effects of higher order cognition induced by a specific task. Such a free viewing paradigm has been used by Maner et al. (2003), where participants had been instructed to naturally view an array of eight faces (half attractive, half average) and by Leder, Tinio, Fuchs, and Bohrn (2010), and Mitrovic, Tinio, and Leder (2016) who presented photos of street scenes showing two people standing next to each other. Both studies found a general effect for attractiveness, in that the attractive faces were looked at longer than the less attractive faces.

In all the studies to date, attractiveness was manipulated as a dichotomous variable, comparing less (or average) attractive faces with more attractive faces. Such predetermined differences hardly reflect the natural variation of attractiveness in everyday encounters. Therefore, in this study, people were unsystematically selected and combined, which resulted in a natural range of differences in facial attractiveness, from subtle to distinct. This approach allows testing if there is a linear relation between the attractiveness of a face and gaze behavior. We expected that the higher the attractiveness of a face, the longer and the more often it would be looked at. Additionally, regarding the sex of the perceiver, we expected the relation between attractiveness and gaze duration to be more pronounced for other sex faces as compared with same sex faces. We furthermore implemented an item to test for effects of the relationship status, expecting the relation between attractiveness and gaze behavior to be less pronounced for participants committed to a romantic relationship (Maner et al., 2003).

To confirm the utility of the aesthetic sense in natural settings—namely guiding visual exploration—faces should be embedded in images of natural environments, in contrast to the often-applied approach of presenting them in an isolated fashion (e.g., Maner et al., 2003; Shimojo et al., 2003). We thereby took the approach by Leder et al. (2010) one step further and presented color photos of natural urban scenes, showing two people, displaying natural differences in attractiveness.

## Method

### Participants

A total of 45 undergraduate students (23 women; mean age *M* = 24.73 years, *SD* = 3.08, age = 20–30 years) took part in exchange for course credit. All participants had normal or corrected to normal vision.

### Materials

**Figure 1.**
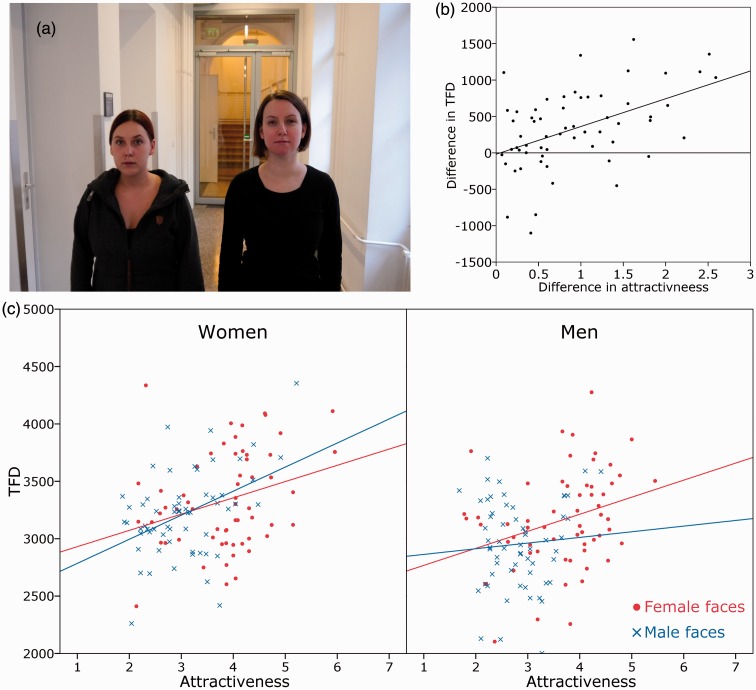
(a) Reenacted example of a scene. (b) Scatterplot illustrating the linear relation between the difference in attractiveness of the two faces and the difference in TFD. (c) Scatterplots illustrating the main effect for attractiveness and the Attractiveness × Sex of Face × Sex of Participant interaction. Mean attractiveness ratings were used instead of single values for illustrative purposes.

Sixty photos of natural, urban real world scenes depicting two people were taken (see Figure 1(a)). A total of 120 people (half women; age 20–30 years) were recruited as models, either by approaching them on the street or by inviting them online. For the actual photographs, all models wore dark clothing, no jewelry, no face piercings, and no heavy makeup; they tied up their hair and removed their glasses if necessary. Dyads were unsystematically formed, resulting in 20 female–female, 20 male–male, and 20 female–male scenes. Each scene was set at a different location in the buildings and the surroundings of the University of Vienna. The models were standing next to each other and were instructed to look straight into the camera, showing a neutral facial expression. Two photos were taken of each of the 60 dyads, with the models switching positions between both versions. The photos were taken at a distance of 2 m, using a Canon EOS 500D (ISO 400, 35 mm), mounted on a camera tripod. Horizontal position of the faces was constant for all photos, roughly dividing the photo in an equally sized left, middle, and right part. Each photo was rescaled to 900 pixels (19.69°) × 600 pixels (13.2°).

For each face, individual, elliptical areas of interest (AOI) were created, covering the face and the hair. The AOIs therefore varied in size, ranging in height between 117 pixels (2.59°) and 213 pixels (4.70°) (*M* = 166 pixels, 3.68°), and in width between 94 pixels (2.08°) and 157 pixels (4.47°) (*M* = 125 pixels, 2.77°). The area covered by the AOIs (AOI-size) ranged from 8913 pixels to 26018 pixels (*M* = 16486 pixels, i.e., 3.05% of the whole image).

### Setting or Apparatus

The experiment was conducted at the University of Vienna in a dimly lit room using a desktop-mounted eye tracker (EyeLink 1000, SR Research Ltd., Experiment Builder software Version 1.10.165, Windows XP, sampling 1000 Hz). While viewing was binocular, the eye tracker was aligned to track the dominant eye. The scenes were presented in a distance of 70 cm on a LCD monitor (Samsung SyncMaster 2443BW, 24″, 1920 × 1200 pixels, 60 Hz).

### Procedure

Participants gave written consent and indicated their sex, age, and relationship status via paper-pencil. Then their visual acuity, oculomotor dominance, and color vision were assessed. Participants rested their head on a chin and forehead rest, and the eye tracker was calibrated and validated (9-point). In the first block, one scene after another was presented for 10 s in random order, while participants were instructed to freely view the images without any required decision or overt task (cf. Maner et al., 2003). Each participant saw one version of each scene, counterbalanced across participants, 60 scenes in total. Between each scene, a fixation cross appeared in the center of the screen, which participants were required to fixate before the next trial began. In case of a drift in the eye tracking signal, the sequence was interrupted and the eye tracker was recalibrated (5-point). In the second and third block, each scene was presented again in random order. In the second block, participants rated the attractiveness of both faces on a 7-point Likert scale ranging from 1 (*very unattractive*) to 7 (*very attractive*). The order in which participants were supposed to rate the two faces randomly changed from trial-to-trial, a small dot beneath the image indicated this. In the third block, each participant indicated the familiarity of each face (*familiar* or *unfamiliar*). The experiment was approved by the ethics board of the University of Vienna and the Austrian Science Fund (FWF project number P27355).

## Results

### Eye Tracking Measures

Three eye tracking parameters were analyzed: (a) total fixation duration (TFD), that is the total duration of all fixations per face in milliseconds, (b) fixation count (FC), that is the total number of fixations per face, and (c) first fixation duration (FFD), that is the duration of the first fixation per face in milliseconds. From the 48,308 cases of the fixation report, 3,103 were removed. Fixations were removed either when they were shorter than 100 ms (Eyelink 1000 user manual, version 1.5.2, SR Research Ltd.), when participants indicated that they were familiar with at least one face, or due to failure of data recording. The average TFD was *M* = 3,169 ms (*SD* = 1,326, *Mdn* = 3,084, *Min* = 105, *Max* = 8,687). Comparing the combined TFD for both faces (6,338 ms) with the overall fixation duration of each trial (8911 ms) showed that faces were looked at for 71% of the time, although their areas covered only 6.1% of the whole images (Birmingham, Bischof, & Kingstone, 2007; Fletcher-Watson, Findlay, Leekam, & Benson, 2008; Hewig, Trippe, Hecht, Straube, & Miltner, 2008; Humphrey & Underwood, 2010). The average FC was *M* = 8.56 (*SD* = 3.55, *Mdn* = 8, *Min* = 1, *Max* = 24). Since FFD (*M* = 320 ms, *SD* = 241, *Mdn* = 236, *Min* = 100, *Max* = 2,671) showed a pronounced right-skewed distribution, a log_10_-transformation was performed (*M* = 2.42, *SD* = 0.25, *Mdn* = 2.37, *Min* = 2, *Max* = 3.43).

### Effects of Attractiveness

TFD, FC, and FFD were analyzed by running linear mixed models using the lme4 package (Version 1.1-8, Bates, Mächler, Bolker, & Walker, 2015) in R (Version 3.1.0, R Development Core Team) applying Satterthwaite approximation for *p* values. Attractiveness was included as a continuous, centered fixed factor. A contrast (male minus female) was included for sex of face and sex of participant. Because the largest AOI was nearly 3 times bigger than the smallest, AOI-size was also included as a continuous, centered fixed factor. Interactions between all factors were included, and the random effect structure justified by the data was maximized. Random by-face intercepts and slopes for attractiveness and sex of participant (TFD and FC only) and random by-participant intercepts and slopes for attractiveness and sex of face were included. No other random slopes or interactions were included, as otherwise the models failed to converge. Also, no random effects for the scenes were included because they explained nearly no additional variance and had no significant effect on the results. By plotting and inspecting the residuals for all models, no violations of linearity, homoscedasticity, or normality were detected. The correlations (*r* in absolute values) of fixed effects were generally low, *M* = .08, *SD* = .11, *Mdn* = .03, *Min* < .01, *Max* = .46.

In general, the same pattern was found for TFD and FC, with a main effect for attractiveness, β = 110, *SE* = 24, *t*(67) = 4.67, *p* < .001 (TFD), β = 0.23, *SE* = 0.06, *t*(72) = 3.7, *p* < .001 (FC), a main effect for sex of face, β = 153, *SE* = 72, *t*(121) = 2.13, *p* = .036 (TFD), β = 0.52, *SE* = 0.2, *t*(134) = 2.82, *p* = .005 (FC), and a three-way Attractiveness × Sex of Face × Sex of Participant interaction, β = −195, *SE* = 52, *t*(1283) = −3.74, *p* < .001 (TFD), β = −0.36, *SE* = 0.14, *t*(3056) = −2.56, *p* = .011 (FC). Additionally, TFD showed an Attractiveness × Sex of Face Interaction, β = 67, *SE* = 32, *t*(133) = 2.09, *p* = .039, showing that the relation between attractiveness and TFD is more pronounced for female faces than for male faces. AOI-size significantly predicted the outcome of all three parameters, β = 0.03, *SE* = 0.01, *t*(191) = 2.54, *p* = .012 (TFD), β < 0.01, *SE* < 0.01, *t*(188) = 5.39, *p* < .001 (FC), β < 0.01, *SE* < 0.01, *t*(608) = −2.27, *p* < .023 (FFD). However, it was not positively confounded with the attractiveness of the faces; collapsed over both sexes, we found a small negative correlation, *r* = −.27, *p* = .002, which simply reflects that female faces are statistically more attractive but cover a smaller area of the scene. Importantly, separated for female and male faces, AOI-size showed no correlation with attractiveness (female faces: *r* = −08, *p* = .53, male faces: *r* = .003, *p* = .98). There were no other significant effects or interactions (all *p*s ≥ .13; see Table 1 for complete results). Figure 1(c) shows the main effect of attractiveness on TFD: The higher a face was rated, the longer it was looked at. Additionally, the four regression lines illustrate a positive effect of other sex: The relation was stronger when women looked at male faces, *b* = .43, *p* < .001, as compared with female faces*, b* = .29, *p* = .026, and also when men looked at female faces, *b* = .31, *p* = .017, as compared with male faces, *b* = .08, *p* = .56. Additionally, the difference in attractiveness of the two faces for each scene was plotted against the difference in TFD. Figure 1(b) shows a positive, linear correlation, *r* = .48, *p* < .001: The bigger the difference in attractiveness, the larger the difference in TFD.

**Table 1.** Fixed Effects for All Eye Tracking Parameters.

	β	*SE*	*df*	*t*	*p*
Total fixation duration (TFD)
Intercept	3,174	101.5	52	31.29	<.001
Attractiveness	110	23.58	68	4.67	<.001
Sex of face (male minus female)	153.2	72.07	121	2.13	.036
Sex of participant (male minus female)	238.2	194.1	44	1.2	.23
AOI-size	0.03	0.01	191	2.54	.012
Attractiveness: Sex of face	66.91	32.01	133	2.09	.039
Attractiveness: Sex of participant	20.95	42.44	51	0.49	.62
Sex of face: Sex of participant	−110.4	81.79	61	−1.35	.18
Attractiveness: AOI-size	<0.01	<0.01	154	−0.02	.98
Sex of face: AOI-size	0.03	0.02	191	1.38	.18
Sex of participant: AOI-size	0.04	0.01	992	0.29	.77
Attractiveness: Sex of face: Sex of participant	−195.2	52.15	1,283	−3.74	<.001
Attractiveness: Sex of face: AOI-size	<0.01	0.01	154	−0.98	.33
Attractiveness: Sex of participant: AOI-size	−0.01	<0.01	3,563	−1.11	.27
Sex of face: Sex of participant: AOI-size	0.02	0.03	988	0.63	.53
Attractiveness: Sex of face: Sex of participant: AOI-size	<0.01	0.02	3,580	−0.47	.64
Fixation count (FC)
Intercept	8.61	0.27	52	31.73	<.001
Attractiveness	0.23	0.06	72	3.7	<.001
Sex of face (male minus female)	0.52	0.19	134	2.82	.005
Sex of participant (male minus female)	0.8	0.52	44	1.53	.13
AOI-size	<0.01	<0.01	188	5.39	<.001
Attractiveness: Sex of face	0.11	0.09	152	1.24	.22
Attractiveness: Sex of participant	<0.01	0.11	52	0.43	.67
Sex of face: Sex of participant	−0.06	0.21	269	−0.29	.77
Attractiveness: AOI-size	<0.01	<0.01	164	0.36	.72
Sex of face: AOI-size	<0.01	<0.01	188	1.44	.15
Sex of participant: AOI-size	<0.01	<0.01	971	1.17	.24
Attractiveness: Sex of face: Sex of participant	−0.36	0.14	3,056	−2.56	.011
Attractiveness: Sex of face: AOI-size	<0.01	<0.01	163	−1.52	.13
Attractiveness: Sex of participant: AOI-size	<0.01	<0.01	3,633	−0.4	.69
Sex of face: Sex of participant: AOI-size	<0.01	<0.01	968	−0.17	.86
Attractiveness: Sex of face: Sex of participant: AOI-size	<0.01	<0.01	3,651	−0.16	.55
First fixation duration (FFD; log_10_)
Intercept	2.42	0.01	47	222.83	<.001
Attractiveness	<0.01	<0.01	67	0.47	.64
Sex of face (male minus female)	<0.01	<0.01	233	0.16	.87
Sex of participant (male minus female)	0.02	0.02	46	1.07	.29
AOI-size	<0.01	<0.01	608	−2.27	.023
Attractiveness: Sex of face	<0.01	<0.01	199	1.12	.25
Attractiveness: Sex of participant	<0.01	<0.01	72	−0.05	.96
Sex of face: Sex of participant	0.02	0.02	323	0.95	.34
Attractiveness: AOI-size	<0.01	<0.01	181	0.84	.4
Sex of face: AOI-size	<0.01	<0.01	610	−0.69	.49
Sex of participant: AOI-size	<0.01	<0.01	5,179	−0.13	.89
Attractiveness: Sex of face: Sex of participant	<0.01	0.01	3,656	0.84	.4
Attractiveness: Sex of face: AOI-size	<0.01	<0.01	181	−0.09	.93
Attractiveness: Sex of participant: AOI-size	<0.01	<0.01	5,216	0	.99
Sex of face: Sex of participant: AOI-size	<0.01	<0.01	5,187	0.12	.91
Attractiveness: Sex of face: Sex of participant: AOI-size	<0.01	<0.01	5,211	0.02	.98

### Leftward Bias

In line with previous research (Ossandon, Onat, & Konig, 2014), a pronounced leftwards bias for the first fixation was found, in that the left face was fixated first in 82% of the cases, χ^2^(1, *N* = 2,622) = 1102, *p* < .001. In comparison, attractiveness did not affect which face was looked at first, as the less attractive face was fixated first in 50.19% of the cases, χ^2^(1, *N* = 1,883) = 0.03, *p* > .75. Importantly, this initial leftwards bias had no effect on the attractiveness ratings or the TFD: mean attractiveness of the left face (*M* = 3.35, *SD* = 0.64) did not differ from the right face (*M* = 3.34, *SD* = 0.67), *t*(59) = 0.16, *p* = .87, and mean TFD for the left face (*M* = 3,170, *SD* = 257) did not differ from the right face (*M* = 3,168, *SD* = 241), *t*(59) = 0.06, *p* = .95.

### Effects of Sex

In a separate analysis, only those scenes were analyzed which depicted a female and a male face. Four analyses of variance were conducted, with attractiveness and the eye tracking parameters as dependent variables, sex of face as a within-participants factor, and sex of participant as a between-participants factor. For attractiveness, TFD, and FC, significant main effects were found for sex of face, *F*(1, 43) = 65.17, *p* < .001, $\eta_{\text{p}}^{2}$^ ^= .6, *d* = 1.06 (attractiveness); *F*(1, 43) = 24.09, *p* < .001, $\eta_{\text{p}}^{2}$^ ^= .36, *d* = 0.59 (TFD); *F*(1, 43) = 25.76, *p* < .001, $\eta_{\text{p}}^{2}$^ ^= .38, *d* = 0.53 (FC), but no main effects for sex of participant, *F*(1, 43) ≤ 3.02, *p* ≥ .09, $\eta_{\text{p}}^{2}$^ ^≤ .07, and no significant Sex of Face × Sex of Participant interactions, *F*(1, 43) ≤ 0.51, *p* ≥ .48, $\eta_{\text{p}}^{2}$^ ^≤ .01. No significant effects were found for FFD (all *p*s ≥ .13). Thus, in the mixed sex scenes, both women and men rated female faces more attractive (*M* = 3.79, *SD* = 0.64) than male faces (*M* = 3.06, *SD* = 0.74) and looked longer and more often at female faces (TFD: *M* = 3,414, *SD* = 731; FC: *M* = 8.7, *SD* = 1.75) than at male faces (TFD: *M* = 2,970, *SD* = 765; FC: *M* = 8.43, *SD* = 1.79).

### Effects of Relationship Status

Finally, effects of the participant’s relationship status on attractiveness and TFD were tested. Twenty-five participants (12 female) indicated to be currently committed to a romantic relationship, 17 (9 female) indicated being single, and 3 (2 female) gave no response. As a trend, participants who were committed to a romantic relationship rated the faces as more attractive (*M* = 3.45, *SD* = 0.6) than single participants (*M* = 3.09, *SD* = 0.56), *t*(40) = 1.95, *p* = .059. Moreover, the relation between the face’s attractiveness and TFD was stronger for single participants, *b* = .37, *t*(118) = 4.38, *p* < .001, than for participants committed to a romantic relationship, *b* = .25, *t*(118) = 2.84, *p* = .005, although the difference between the coefficients was not significant, *t*(236) = .92, *p* = .36.

## Discussion

Positive effects of facial attractiveness on gaze behavior have already been found in previous studies (e.g., Leder et al., 2010; Maner et al., 2003; van Straaten, Holland, Finkenauer, Hollenstein, & Engels, 2010). However, up to now, all these studies compared gaze behavior in respect of more attractive faces versus faces which had been preselected to be clearly less attractive. Such a predefined difference between two faces is not very representative for everyday life, where we encounter all combinations and variations of attractiveness. Therefore, instead of comparing more with less attractive faces, we unsystematically selected and combined two people and created scenes naturally in regard of differences in facial attractiveness. These scenes were presented in a free viewing eye tracking paradigm. We found a clear effect of facial attractiveness, in terms of a sensitive and linear function of the aesthetic sense in guiding visual attention: The more attractive a face was rated, the longer and the more often it was looked at. This relation was also reflected in comparing the two faces of each scene: The larger the difference in attractiveness, the larger the difference in TFD. Figure 1(b) shows that for most scenes, already a relatively small difference in attractiveness was sufficient to enhance gaze duration in favor for the more attractive face. Our study is correlational so causal conclusions are not warranted. It is also possible that enhanced gaze duration influences the attractiveness ratings (Little, DeBruine, & Jones, 2013; Zajonc, 1968). Shimojo et al. (2003) called this interaction a “gaze cascade effect,” a circular enhancement in that attractiveness leads to longer gaze duration, which increases attractiveness in turn. It would be interesting for future studies to estimate the relative share of attractiveness and gaze duration by experimentally manipulating causal directions.

Moreover, we found that the guidance of facial attractiveness on visual exploration was more pronounced for other sex faces. This result therefore is in line with the “opposite-sexed beauty captures the mind” hypothesis (Maner et al., 2003) as well with the idea that there is an evolutionary link between facial attractiveness and visual attention (e.g., Rhodes, 2006). This relation also applied to women looking at male faces, which is in contrast to findings by van Straaten et al. (2010) who only found an effect for men who looked at female faces. The contradictory results might reflect a difference between passively viewing faces in the laboratory and real-life settings, in which women might use different mating strategies. Nevertheless, besides the more pronounced relation for other sex faces, the linear relation also applied to women looking at female faces, and the mixed sex scenes revealed that female faces—which were statistically more attractive than male faces—were looked at longer independent from the sex of the perceiver. These two findings cannot simply be explained by sexually driven behavior but imply a more general role of attractiveness in guiding visual attention. The picture is more complicated for men looking at male faces, which only showed a weak linear relation. This weak relation might also explain the finding that the relation between attractiveness and TFD is slightly stronger for female faces than for male faces. Looking closer at the male faces (blue dots) for men in Figure 1(c) might indicate why it seems that male faces show a u-shaped distribution in that less attractive faces display a negative correlation, whereas more attractive faces positively correlate with fixation duration. The small range in attractiveness for male faces did not justify testing nonlinear relations in this study, but we suggest that future research considers nonlinear relations, in order to gain a more comprehensive picture of sex-related interactions.

Additionally, we found that the relation between attractiveness and gaze duration was larger for single participants than for participants committed to a romantic relationship (Maner et al., 2003). This is in line with the idea that romantically involved people generally seem to react less sensitive to attractive faces in many ways (Karremans & Verwijmeren, 2008; Maner, Gailliot, Rouby, & Miller, 2007; Maner, Rouby, & Gonzaga, 2008; Simpson, Gangestad, & Lerma, 1990). We also found that single participants rated the faces generally less attractive than participants committed to a relationship, which is contrary to previous findings by Simpson et al. (1990). The different results might have occurred because of differences in the design, as Simpson et al. (1990) used a combination of attractiveness and sex appeal as dependent variables, as well as highly attractive faces. We therefore argue that future research should consider different aspects of facial attractiveness measures (Dai, Brendl, & Ariely, 2010), as well as considering different psychological processes for faces of relatively high attractiveness. Together, the interpersonal differences reflect the importance of motivational aspects moderating the effects of attractiveness on visual attention (Leder et al., 2010).

To sum up, we found a general and sensitive effect of facial attractiveness in guiding visual exploration in natural scene perception. The more attractive the face, the longer it was looked at. This study is the first to show that this relation is not restricted to very attractive faces, or faces clearly differing in attractiveness, but applies to faces and all levels of attractiveness in general.
